# Microbial signatures in head and neck squamous cell carcinoma: an *in silico* study

**DOI:** 10.1590/1678-7757-2024-0392

**Published:** 2025-02-03

**Authors:** Loganathan KAVITHA, Manogaran KUZHALMOZHI, Jayaseelan VIJAYASHREE PRIYADHARSINI, Arunachalam ARUN KUMAR, Krishna Mohan Rao UMADEVI, Kannan RANGANATHAN

**Affiliations:** 1 The Tamil Nadu Dr. MGR Medical University Ragas Dental College and Hospital Department of Oral and Maxillofacial Pathology Chennai India The Tamil Nadu Dr. MGR Medical University, Ragas Dental College and Hospital, Department of Oral and Maxillofacial Pathology, Chennai, India.; 2 Aringnar Anna Memorial Cancer Research Institute Chennai India Aringnar Anna Memorial Cancer Research Institute, Chennai, India.; 3 Saveetha Institute of Medical and Technical Sciences Saveetha University Saveetha Dental College & Hospital Chennai India Saveetha Institute of Medical and Technical Sciences, Saveetha University, Saveetha Dental College & Hospital, Chennai, India.; 4 Independent Researcher Hosur India Independent Researcher, Hosur, India.

**Keywords:** Microbiome, In silico, Oral squamous cell carcinoma, HNSCC, UALCAN, TCMA

## Abstract

**Objectives:**

The oral cavity harbors a plethora of bacterial species. Dysbiosis of oral and gut microbiota is associated with several oral and systemic pathologies, such as cancer, obesity, diabetes, atherosclerosis and gastrointestinal diseases. Imbalance in the oral-gut microbial axis has been associated with head and neck squamous cell carcinoma (HNSCC). This study aims to analyze the bacterial profile of HNSCC across various taxonomic units, investigate molecular patterns associated with prevalent bacterial phylum in HNSCC, and compare the bacterial profile in HNSCC and gastrointestinal (GI) carcinoma using computational analysis.

**Methodology:**

The microbe-host transcriptomic, proteomic, and epigenetic analyses of HNSCC and GI carcinomas were performed using The Cancer Microbiome Atlas (TCMA) database. The differential expression of the host’s mRNA transcripts and proteins associated with tumor microbiome were analyzed using The University of Alabama at Birmingham Cancer data analysis (UALCAN) and Clinical Proteomic Tumor Analysis Consortium (CPTAC) websites.

**Results:**

A decrease in Actinobacteria and an enrichment of Flavobacteria at the class level, Neisseriales, Pasteurellales, and Campylobacterales at the order level, *Pasteurellaceae, Flavobacteriaceae, Campylobacteraceae*, and *Peptoniphilaceae* at the family level, and *Hemophilus, Porphyromonas*, and *Leptotrichia* at the genus level were observed in HNSCC compared to the normal mucosa. RICTOR protein, mRNA transcripts (HIST1H2BB, SCARNA11, TBC1D21 gene), and hsa-miR-200a-5p miRNA were significantly correlated with prevalent bacterial species in HNSCC. A major increase in Actinobacteria, Fusobacteria, and Spirochaetes was observed in HNSCC compared to GI carcinoma.

**Conclusion:**

The oral-gut microbial dysbiosis, as reflected by the differential abundance of bacterial species in oral and GI carcinomas, suggests the implication of tumor microbiome and their genomic interactions with the host in carcinogenesis.

## Graphical abstract

**Figure:**
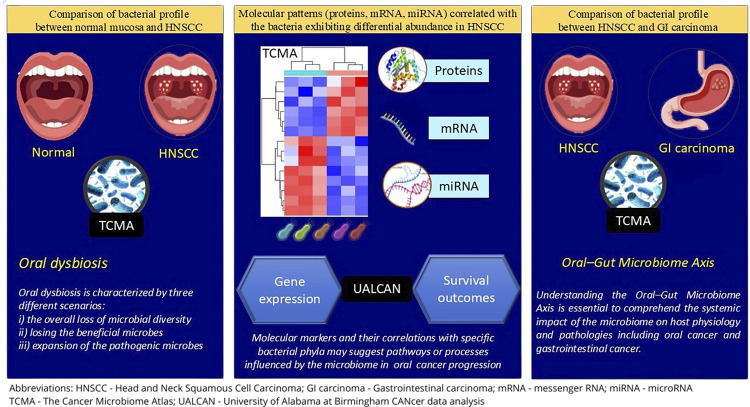

## Introduction

The oral microbiome comprises bacteria, fungi, viruses, archaea, and protozoa living in the oral cavity. Approximately 700 bacterial species have been identified, making it the second-largest bacterial community with the second-highest level of alpha diversity (microbial diversity within individuals) in the human body, following the gut.^1^In a healthy host, the oral microbiome maintains balanced symbiotic/commensal relationships, described as ‘‘microbial homeostasis” or ‘‘eubiosis.” This promotes beneficial commensalism without harming the microorganisms or the host. It is also essential in the development of natural oral physiology and defense mechanisms. Inter-species and host-microbial interactions regulate the microbial composition and can impact the health of the host.^2^The oral microbiome can shift this balance from commensalism to an unbalanced parasitic/pathogenic disease state, known as an ‘‘unbalanced microbiome” or ‘‘dysbiosis”.^1,2^The shift in the oral microbiota from eubiosis to dysbiosis can provoke various pathological conditions, including carcinogenesis.^2^

Dysbiosis is characterized by loss of microbial diversity, loss of beneficial microbes, and expansion of the pathogenic microbes as an isolated or simultaneous process,^1,3^being associated with several intraoral and systemic diseases. The role of microorganisms in the former, such as dental caries, gingivitis, periodontitis, and oral candidiasis, is well established. The number of microorganisms ingested daily is about one hundred billion (10^11^) to one trillion (10^12^). The oral microbiota has unrestricted access to the gastrointestinal tract and other organ systems, which explains its impact on systemic diseases such as Alzheimer’s disease, diabetes, pregnancy complications, and several types of cancer, including oral, gastrointestinal, lung, breast, prostate, and uterine cancer.^2,4^ Oral microbiota-mediated carcinogenesis includes sustaining proliferative signaling, the ability to evade growth suppressors and induce angiogenesis, resisting cell death, limitless replicative potential, and activation of invasion and metastasis.^4^

Head and neck squamous cell carcinoma (HNSCC) varies worldwide and is generally correlated with tobacco-derived carcinogens and excessive alcohol consumption. Oral squamous cell carcinoma (OSCC) constitutes around 90% of HNSCC and is often preceded by oral potentially malignant disorders (OPMDs) with varying risks of malignant transformation to OSCC. Oral cancer is the 16^th^ most common malignancy, accounting for 389,485 new cases, and is the 15^th^ leading cause of mortality globally.^5^ Oral bacteria community dynamics have been linked to OSCC staging in studies, raising the possibility of using bacteria as OSCC diagnostic markers. Salivary microbiome dysbiosis and cytokines influence OSCC through inflammation.^6^*Fusobacterium nucleatum, Porphyromonas gingivalis,* and *Prevotella intermedia* are strongly associated with OSCC; other bacterial genera demonstrated in OPMDs and OSCC include *Actinomyces, Clostridium, Enterobacteriaceae, Fusobacterium, Haemophilus, Porphyromonas, Prevotella, Streptococcus spp. (S. infantis, S. mitis, S. gordonii)* and *Veillonella*. Several periodontal disease-associated bacterial species, namely *Porphyromonas gingivalis, Tannerella forsythia,* and *Prevotella intermedia*, are correlated with an increased risk of gastrointestinal (GI) cancer.^7^

Characterizing oral microbiome is essential to understand oral health and systemic diseases. *In silico* studies serve as prediction models and can give insight to initiate experimental studies. Recently, *in silico* studies analyzed the mRNA expression and mutation profiles of the target genes in human cancer, including HNSCC, colorectal, pancreatic, hepatic, and lung cancers.^8-12^Using *in silico* analysis, we investigated the microbial profile of oral bacteria in HNSCC across different taxonomic units, the molecular patterns (differential expression of protein, mRNA, microRNA, and DNA methylation sequences) in the host associated with the oral bacteria during carcinogenesis, and compared the bacterial prevalence in HNSCC and GI carcinoma to increase the understanding of the oral-gut microbial axis in carcinogenesis. This study also discusses the role of oral dysbiosis in the xenobiotic metabolism of carcinogens, which are crucial in the development of OPMDs and OSCC.

## Methodology

### Data source

Computation (*in silico*) analysis unraveling the bacterial profile and molecular signatures in HNSCC employs two comprehensive, user-friendly, and interactive websites: The Cancer Microbiome Atlas (TCMA) and the University of Alabama at Birmingham Cancer database (UALCAN).^13,14^

TCMA is a database of decontaminated and tissue-resident microbial profiles of The Cancer Genome Atlas (TCGA). It provides a collection of curated microbial compositions of oropharyngeal, esophageal, gastrointestinal, and colorectal tissues in healthy controls and carcinoma. It is a resource for performing multi-omic, pan-cancer analyses of host-microbe interactions that enable the identification of diagnostic and prognostic species. TCMA facilitates a matched microbe-host transcriptomic, proteomic, and epigenetic analysis that identifies associations between microbes and gene expression patterns and pathways of the host.^13^

UALCAN is a website that enables researchers to analyze gene expression using the TCGA database on about 20,500 protein-coding genes in 33 different tumor types. It provides interactive graphs and plots depicting gene expression profiles and their influence on patient survival.^14^

### Study workflow and selection criteria

In this study, the TCMA data repository was used to analyze the prevalence of bacteria in HNSCC and its association with the host molecular characteristics. The prevalence of bacteria in HNSCC was compared to that of normal tissue in the following taxonomic units: phylum, class, order, family, and genus.

The phyla predominant in HNSCC were correlated with the host molecular characteristics, which include RPPA (reverse-phase protein array) proteins, mRNA and miRNA sequences. Interactive heat maps showed the correlation between each bacterial phyla and the host molecular features. The molecular traits (proteins/mRNA/miRNA) with the highest and lowest correlation values with each bacterium were screened. RPPA proteins, mRNA, and miRNA sequences that demonstrated a moderate and strong correlation with the bacterial phyla in the TCMA database were selected. Their gene expression and the effect of the gene on HNSCC survival outcomes were studied in the UALCAN database.

TCMA also facilitated the comparison between the relative abundance of bacteria in HNSCC and GI carcinoma (esophageal carcinoma, adenocarcinoma, colon adenocarcinoma, and rectal adenocarcinoma).

The proportion test was done to compare various microbiomes in tumor tissue and normal tissue using STATA (ver.12), with p<0.05 representing statistical significance. Oral microbial dysbiosis involving xenobiotic metabolism in OPMDs and oral and gastrointestinal carcinogenesis were reviewed from the literature. The study workflow is summarized in the graphical abstract.

## Results

The TCMA database included microbiome compositions of 177 HNSCC samples (155 tumors and 22 tumor-adjacent tissue as normal samples) at phylum, order, and genus levels. Table 1 summarizes demographic details of the samples. The TCMA database was used to extract 11 phyla, 38 orders, and 221 genera of microbial taxa in each sample.

**Table 1:** Clinicopathological characteristics of normal mucosa and HNSCC tissue samples

Clinicopathological characteristics	Normal	HNSCC
**Gender [(n (%)]**		
Male	15 (68.2)	113 (72.9)
female	7 (31.8)	42 (31.8)
Age (median range)	60 (29-87)	60 (19-90)
**Site of biopsy [n (%)]**		
Tongue base	1 (4.5)	7 (4.5)
Mouth floor	0 (5.2)	8 (5.2)
larynx	2 (9.1)	22 (14.2)
**Tumor stage [n (%)]**		
Stage I	-	10 (6.5)
Stage II	-	23 (14.8)
Stage III	-	23 (14.8)
Stage IV	-	73 (47.1)
Not reported	-	26 (16.8)
**Histologic grade [n (%)]**		
Grade 1	-	13 (8.4)
Grade 2	-	93 (60.0)
Grade 3	-	44 (28.4)
Grade 4	-	1 (0.6)
Grade X	-	4 (2.6)
**Total**	**22**	**155**

### Differential bacterial profiling of HNSCC tumor tissue and normal samples (Figure 1)

A relative abundance of obligate, anaerobic, Gram-negative bacilli belonging to the phylum Fusobacteria (Class: Fusobacteriia, Order: Fusobacteriales; Family: *Fusobacteriaceae*, Genus: *Fusobacterium*), and the phylum Bacteroidota (synonym: Bacteroidetes) (Order: Bacteroidales, Family: *Prevotellaceae* and Genus: *Prevotella*) was observed in both HNSCC and normal mucosa. At the order level, Campylobacterales were enriched in the tumor tissue compared to the normal tissue; Veillonellales and Coriobacteriales were significantly abundant in the normal tissue (p<0.05). At the family level, Flavobacteriaceae and Camplyobacteraceae were prevalent in tumor tissue, whereas Neisseriaceae was more prevalent in normal tissue (p<0.05). At the genus level, *Hemophilus, Porphyromonas,* and *Leptotrichia* were significantly recurrent in tumor tissues, whereas *Actinomyces* and *Rothia* were relatively abundant in the adjacent normal tissue (p<0.05). Actinobacteria was lower in tumors compared to normal tissue in all taxonomic units, from phylum to genus (p<0.05).

**Figure 1:**
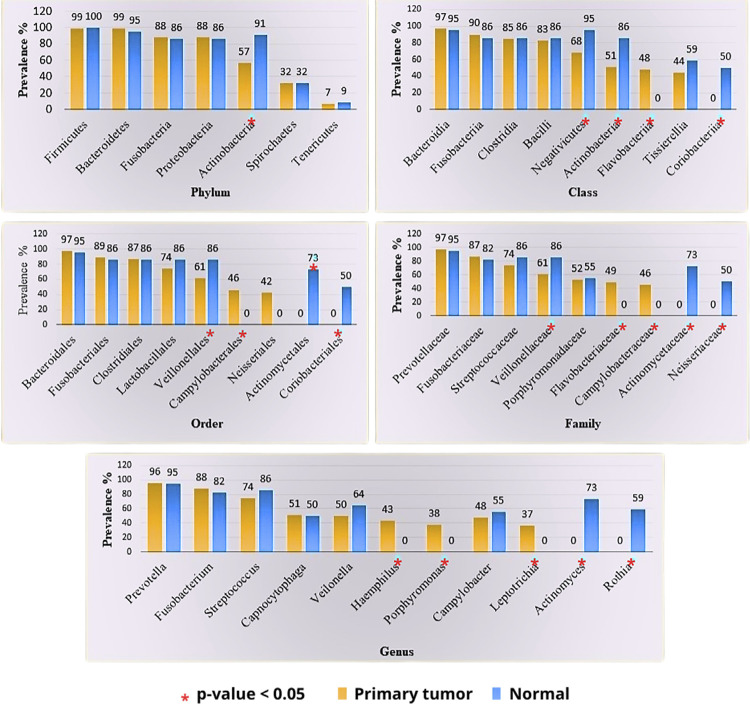
Prevalence of bacteria across various taxonomic units in HNSCC and normal mucosa.

### Molecular patterns (proteins, mRNA, miRNA) correlated with bacteria exhibiting differential abundance in HNSCC (Figure 2; Table 2)

RICTOR protein was negatively correlated (r=-0.56; moderate correlation) with Bacteroidetes. The mRNA transcripts of the HIST1H2B gene (r=-0.72; strong correlation), SCARNA11 gene (r=-0.45; moderate correlation), and TBC1D21 gene (r=-0.59; moderate correlation) were negatively correlated with Firmicutes, Proteobacteria, and Bacteroidetes respectively. The hsa-miR-200a-5p showed a moderate positive correlation (r=0.44) with Firmicutes. hsa-miR-365-5p (r=0.25), hsa-miR-374a-5p (r=0.22), and hsa-miR-365b-5p (r=0.20) exhibited a weak positive correlation with Proteobacteria, Bacteroidetes, and Fusobacteria respectively. The hsa-miR-365a-5p (r=-0.35) and hsa-miR-338-3p (r=-0.36) demonstrated a weak negative correlation with Actinobacteria and Proteobacteria in HNSCC, respectively.

**Figure 2:**
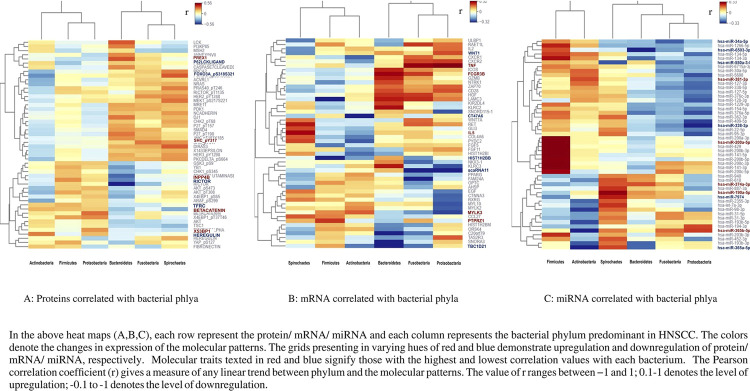
Correlation of bacterial phylum and host molecular features (Proteins, mRNA, miRNA) in HNSCC.

**Table 2:** The molecular patterns (proteins, mRNA, and miRNA) correlated with the bacteria exhibiting differential abundance in HNSCC.

Predominant Phylum		Actinobacteria	Firmicutes	Proteobacteria	Bacteroidetes	Fusobacteria	Spirochaetes
Host RPPA proteins	Positively correlated proteins	X53BP1 r= 0.28	INPP4B r= 0.26	Beta-catenin r= 0.31	PREX1 r= 0.35	SHC_pY317 r= 0.29	FOXO3A r= 0.34
	Negatively correlated proteins	FOXO3A r= - 0.37	P62LCKL ligand r= - 0.26	Heregulin r= - 0.24	RICTOR r= - 0.56	RICTOR r= - 0.28	TFRC r= - 0.31
Host mRNA-seq	Positively correlated mRNA-seq	MYLK3 r= 0.20	PTPRZ1 r= 0.27	TNF r= 0.36	FCGR3B r= 0.30	TNF r= 0.31	IL5 r= 0.29
	Negatively correlated mRNA-seq	CT47A6 r= - 0.33	HIST1H2BB r= - 0.71	scaRNA11 r= - 0.45	TBC1D21 r= - 0.55	scaRNA11 r= - 0.40	WNT1 r= - 0.22
Host miRNA-seq	Positively correlated miRNA-seq	hsa-miR-381-3p r= 0.22	hsa-miR-200a-5p r= 0.44	hsa-miR-365b-5p r= 0.23	hsa-miR-374a-3p r= 0.21	hsa-miR-365b-5p r= 0.19	hsa-miR-190a-5p r= 0.32
	Negatively correlated miRNA-seq	hsa-miR-365a-5p r= - 0.36	hsa-miR-7974 r= - 0.22	hsa-miR-338-3p r= - 0.36	hsa-miR-6503-3p r= - 0.33	hsa-miR-34a-5p r= - 0.27	hsa-miR-550a-3-5p r= - 0.28

**Figure 3:**
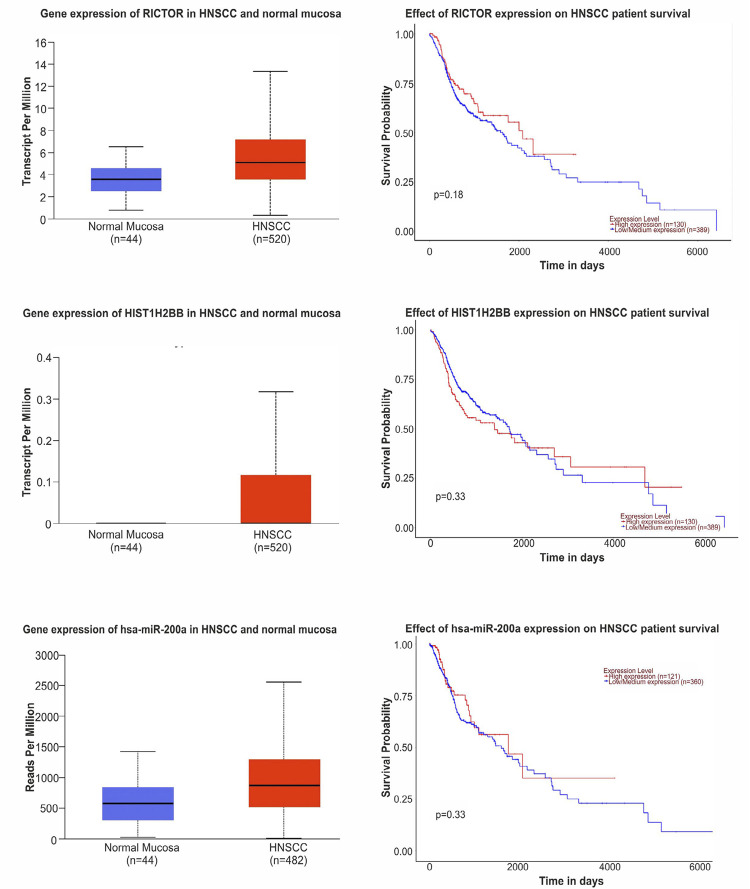
Gene expression of RICTOR, HIST1H2BB, and miR-200a-5p in HNSCC and their effect on survival outcomes.

The UALCAN database demonstrated the gene expression of molecular patterns significantly associated with the bacterial phyla abundant in HNSCC and the effect of differential gene expression (DGE) on HNSCC survival outcomes (Figure 3). Upregulation of RICTOR and HIST1H2B gene was observed in HNSCC (Median: 5.1, 0.1, respectively; transcripts per million) compared to normal mucosa (Median: 3.6, 0, respectively; transcripts per million). Similarly, hsa-miR-200a expression was greater in HNSCC (Median: 872.6; reads per million) compared to normal mucosa (Median: 578.1; reads per million). However, UALCAN had insufficient data on scaRNA11 and TBC1D21 for analysis.

### Comparison of bacterial profile in HNSCC and GI Carcinoma

**Figure 4:**
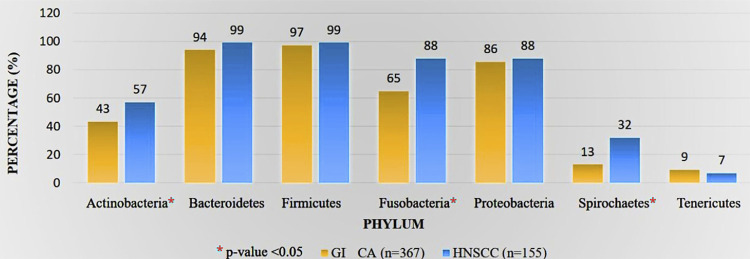
Comparison of bacterial profile between HNSCC and GI carcinoma.

At the phylum level, Actinobacteria, Fusobacteria, and Spirochaetes were increased in HNSCC compared to GI carcinoma (p<0.05) (Figure 4).The prevalence of Bacteroidetes, Firmicutes, Proteobacteria, and Tenericutes did not vary significantly between both groups. *Alistipes, Blautia, Faecalibacterium, Parabacteroides, Prevotella, Roseburia, Ruminococcus, Streptococcus*, Verrucomicrobia, and Thermotogae were enriched in GI carcinoma compared to HNSCC. Supplementary Table 1 summarizes the difference in the bacterial prevalence in other taxonomic units between HNSCC and GI carcinoma.

## Discussion

The human microbiota is a complex, diverse, and abundant population of symbiotic microorganisms that inhabit many sites in the human body, including the skin, oral cavity, and gastrointestinal tract (GIT; gut). The oral cavity is the entry of all microorganisms into the human body through the GIT. The microbiota of the oral cavity and the GIT are the largest microbial ecosystems in the human body with the predominance of the bacterial microbiota. The bacterial diversity of the oral-gut microbial axis and their complex interactions have been implicated in the pathogenesis of oral and gastrointestinal diseases, including cancer development and progression.^15,16^ In this study, we analyzed the bacterial profile involving 11 taxa with the associated host molecular patterns in 155 HNSCC cases and a portion of their adjacent normal tissues from 22 cases. The differential abundance of the bacteria in the oral cavity was compared to that of GIT to understand the role of the oral-gut microbial axis in oral and GI carcinogenesis.

In this study, an abundance of obligate, anaerobic, Gram-negative bacilli included in the Fusobacteria phylum (Class: Fusobacteriia, Order: Fusobacteriales; Family: *Fusobacteriaceae*, Genus: *Fusobacterium*), and the Bacteroidota phylum (synonym: Bacteroidetes) (Order: Bacteroidales, Family: *Prevotellaceae* and Genus: *Prevotella*) was observed in both HNSCC and normal mucosa.

In oral cancer, *F. nucleatum* causes double-stranded DNA breaks and promotes GLUT1 upregulation and lactic acid accumulation. Its lipopolysaccharides can stimulate inflammatory cytokines, leading to a proinflammatory environment and promoting tumor progression.^17^ In colorectal cancer, the Fap2 protein of *F. nucleatum* interacts with the TIGIT receptor (T cell immunoreceptor with Ig and ITIM [immunoreceptor tyrosine-based inhibitory motif] domains) present on natural killer (NK) cells. It inhibits their cytotoxicity and, subsequently, contributes to tumor evasion. The protein Fad A of *F. nucleatum* binds to E-cadherin on colorectal cancer cells and induces β -catenin signaling, leading to regulation of inflammatory and oncogenic responses.^18^*F. nucleatum* regulates the polarization of macrophage to M2 phenotype by secreting IL-6 and activating IL-6/STAT3/c-MYC signaling. *Bacteroides fragilis* induce the production of IL-8 by activating E-cadherin/β-catenin/NF-kB signaling pathway.^19^*Bacteroides fragilis* toxin (BFT) secreted by *B.fragilis* degrades E-calmodulin, causing alterations in signaling pathways that lead to upregulation of spermidine oxidase, which in turn promotes irreversible DNA damage and may eventually lead to carcinogenesis.^20^*P. intermedia* treatment significantly stimulates tumor growth, invasion, angiogenesis, and metastasis, affects levels of inflammatory cytokines, and alters M2 macrophages and regulatory T cells (Tregs) infiltration in the tumor microenvironment. It stimulates tyrosine kinase receptors that modulate cell proliferation, migration, and differentiation associated with disease progression.^21^

**Figure 5:**
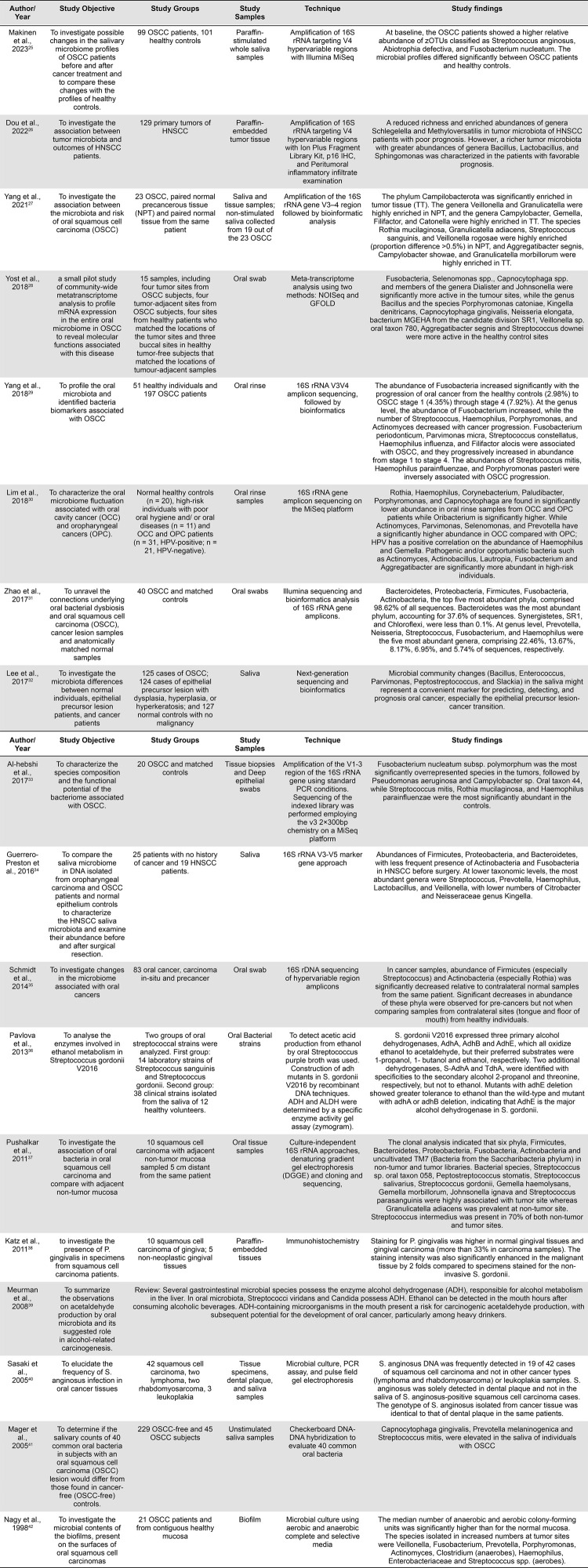
The differential abundance of the oral microbiota across various taxonomic units in HNSCC and OPMD.

We also observed an enrichment of Flavobacteria at the class level, Neisseriales, Pasteurellales, and Campylobacterales at the order level, *Pasteurellaceae, Flavobacteriaceae, Campylobacteraceae*, and *Peptoniphilaceae* at the family level, and *Hemophilus, Porphyromonas*, and *Leptotrichia* at the genus level were observed in HNSCC compared to the normal mucosa. *Pasteurella multocida* produces a toxin that modifies heterotrimeric G-proteins and activates Rho GTPase, focal adhesion kinase, cyclooxygenase-2, β-catenin signaling, and calcium signaling pathways involved in carcinogenesis.^22^*Porphyromonas gingivalis* has virulence factors such as gingipains, outer membrane vesicles (OMVs), E-cadherin, toxin, and proteolytic enzymes. *P. gingivalis* can also convert ethanol to acetaldehyde, a carcinogenic intermediate.^23^*Leptotrichia hofstadii* was abundant in stage III oropharynx cancer.^24^ Various studies have explored the differential abundance of the oral microbiota across various taxonomic units. Figure 5 summarizes important observations from some of these studies.^25-42^

An enrichment of Actinobacteria (phylum), Negativicutes, Coriobacteria (class), Veillonellales, Actinomycetales, Coriobacteriales, Micrococcales (order), *Veillonellaceae, Neisseriaceae, Actinomycetaceae, Micrococcaceae* (family), *Actinomyces, Rothia,* and *Atopobium* (genus) in the normal mucosa was observed in this study. This supports the protective role of actinobacteria, which is well documented in the literature.^33,35,43^ Actinobacteria are Gram-positive bacteria with high Guanine and Cytosine content in their genetic makeup. Actinobacteria produce a wide range of natural bioactive compounds and secondary metabolites due to their environmental diversity and metabolic potential. They are diverse groups of microorganisms that are ubiquitous in the aquatic and terrestrial environment. The natural compounds and metabolites from Actinobacteria produce a range of anti-cancer, immunosuppressive, anti-parasitic, and anti-viral agents.^44^ Consistent with the observations of this study, Al-Hebshi, et al.^33^ (2017) and Schmidt, et al.^35^ (2014) report that *Rothia sp*., belonging to the phylum Actinobacteria, is associated with relative abundance in healthy controls and is decreased in OPMDs and OSCC. In contrast with the beneficial role of Actinobacteria, pancreatic head carcinoma is associated with phylum Actinobacteria (*Rothia, Actinomyces, Corynebacterium*).^35^*Neisseria elongate* has been associated with pancreatic cancer. Karpiński (2019)^45^and Muto, et al.^46^ (2000), in an *in vitro* study, reported that *Neisseria* can be a regional source of carcinogenic acetaldehyde and may play an essential role in alcohol-related carcinogenesis in humans. They demonstrated the ١٠٠-fold higher ability of *Neisseria* to produce acetaldehyde compared to *Streptococcus sp., Stomatococcus sp*., or *Moraxella sp*.

The oral microbiome forms a close symbiotic relationship with human host cells in the oral cavity. The molecular patterns in the host substantially influence the differential expression of microorganisms. A negative correlation was observed between the RICTOR protein and Bacteroidetes. The gene and protein expression of RICTOR was upregulated in HNSCC with poor survival outcomes in patients showing high RICTOR expression. The tumor-promoting role of the RICTOR protein and the probiotic nature of Bacteroidetes correlate with the observations of this study. Rapamycin-insensitive companion of mTOR (RICTOR), a subunit of mTOR, is a critical regulator of the PI3K/AKT pathway and plays an essential role in tumors driven by receptor tyrosine kinase (RTK) alterations. Inhibition of RICTOR protein and blocking RTK co-activation can serve as an independent or combined therapeutic target.^47^RICTOR is a scaffold protein for Integrin-Linked Kinase (ILK), a β1-integrin interacting protein with kinase activity. The mTOR-independent ILK/RICTOR complex was detected in several cancer cell lines and is involved in Transforming Growth Factor (TGF) beta-1-mediated EMT.^48^ RICTOR promotes tumor progression regulating the tumoral microenvironment by increasing cell proliferation and survival, decreasing apoptosis in cancer cells, facilitating angiogenesis, and remodeling the stroma. Overexpression of RICTOR is positively associated with tumor progression and poor survival in colorectal cancer, hepatocellular carcinoma (HCC), endometrial carcinoma, pituitary adenoma, and pancreatic ductal adenocarcinoma.^47^ Using next-generation sequencing, it was shown that RICTOR up-regulation strengthens mTORC2 activity, promoting cell growth and motility. Conversely, RICTOR down-regulation suppresses cell proliferation and tumor formation.^49^*Bacteroides*, the predominant genus in the phylum Bacteroidetes, comprise Gram-negative bacteria with a rod-shaped morphology. In healthy adults, *Bacteroides* comprise 20-80% of gut microbiota, constituting the predominant flora. *Bacteroides spp*. possess polysaccharide utilization loci that enable them to metabolize polysaccharides that are not easily absorbed in the intestine, providing energy to adjacent bacteria and helping maintain the balance of gut microbiota.^50^

The mRNA sequence of Histone 1 H2b (HIST1H2B) was negatively correlated with Firmicutes. Oncohistones have emerged as a new field in cancer epigenetics research. Oncohistone mutations are clustered mono-allelic missense mutations that often affect only one of the histone genes (human histones are polygenic; all four histones are encoded by at least 15 genes), the expression of which exhibits oncogenic features. Garciaz, et al.^51^ (2019), reported that HIST1H1D plays a role in acute myeloblastic leukemia blast cell lineage differentiation. Histone 2 mutations have been reported in pancreatic cancer, glioblastoma, prostate cancer, and lung cancer.^52^ Zhao and Dai^53^ (2021) reported that HIST3H2A might regulate the progression of tumor immunity in pancreatic cancer by modulating the JAK-STAT pathway.^53^

Similarly, a negative correlation was observed between the mRNA sequence of scaRNA11 and TBC1D21 and Proteobacteria and Bacteroidetes, respectively. Small Cajal body-specific RNAs (scaRNAs) guide post-transcriptional modification of spliceosomal RNA and have defined roles in tumorigenesis. scaRNA11 is one of the 20 upregulated genes associated with RNA-binding protein HuR in thyroid cancer cells.^54^ Tian, et al.^55^ (2022) report that TBC1 domain family member 2 (TBC1D2) is overexpressed in ovarian cancer and contributes to tumor metastasis via epithelial cadherin (E-cadherin) degradation. However, the role of scaRNAs and TBC1D21 is not elucidated in HNSCC.

Hassan, et al.^8^ (2023) in an *in silico* analysis of DGE in colorectal cancer, identified STAT3 and HNRNPA2B1 as key hub proteins in colorectal cancer (CRC). Raj, et al.^9^ (2024) demonstrated negative correlation between c-MET and immune cell infiltration, suggesting c-MET might have a role in immune suppression in the TCGA HNSCC dataset using different *in silico* tools. Abu-Shahba, et al.^10^ (2023) reported that patients with high expression of POU2F1 or low expression of PPARA exhibited low survival probability and vice versa (p≤0.05) in HCC. Gene expression analysis in lung squamous cell carcinoma showed a significant downregulation of DEL-1 and IL-6 and upregulation of CXCL13, suggesting the role of differential expression of these genes in lung carcinogenesis.^11^ Computational analysis using the Gene Expression Omnibus (GEO) database (GSE172096 dataset) revealed that transmembrane protein (TMEM2), associated with higher-risk groups in pancreatic adenocarcinoma was strongly correlated with familial adenomatous polyposis (FAP) gene, a cancer-associated fibroblast (CAF) marker.^12^

Firmicutes, also known as Bacillota, are Gram-positive bacteria. Zhang, et al.^56^ (2020) and Li, et al.^57^ (2021) report an abundance of Firmicutes in oral cancer and healthy controls, similar to the findings of this study. However, Yang, et al.^29^ (2018) and Guerrero-Preston, et al.^34^ (2016) reported a higher abundance of Firmicutes in the tissue samples and saliva rinses of OSCC patients. The hsa-miRNA-2005p showed a moderate positive correlation with Firmicutes in this study. miRNAs (microRNAs, miRs) are small non-coding RNAs that regulate gene expression at the post-transcriptional level. miRNA dysregulation is often reported in carcinogenesis. miR-200 family members act by targeting several mRNAs associated with cancer cell proliferation.^58^miR-200a targets Cyclin-Dependent Kinase 6 (CDK6) in melanoma and thus causes cell-cycle arrest and decreases cancer cell proliferation. A downregulation of miR-200a and miR-125a has been reported in oral cancer patients. It is considered that a decrease in miR-200a promotes the epithelial-mesenchymal transition (EMT) of tumor cells.^59^

Oral microbiota dysbiosis is associated with oral cancer development by various mechanisms. Inflammatory cytokines and matrix metalloproteinases promote development and progression of tumors. The oral bacteria produce oxygen and nitrogen-reactive species, and oncogenic metabolites (e.g., nitrosamines) induce genetic damage to oral mucosal cells. There is an alteration of the epithelial barriers predisposing to OPMDs. Several epigenetic alterations (e.g., alteration of onco-miR or DNA methylation phenomena) are implicated in oral dysbiosis associated with tumorigenesis.^7^

The use of tobacco, with or without areca nut and alcohol use, has a substantial contribution to oral dysbiosis associated with the development of OPMDs and OSCC. A loss of commensal bacteria with a protective role, such as *Neisseria*, and a reduced response to *Porphyromonas gingivalis* is associated with tobacco use. A decrease in *Lactobacilli*, a commensal of the oral mucosa that can break down salivary acetaldehyde (carcinogen) production, is observed in alcohol consumers.^60^ Amer, et al.^61,62^ (2017, 2020) reported that smokers had reduced levels of *Neisseria sp., Fusobacterium nucleatum*, and *Leptotrichia*, and alcohol consumers showed increase in *Campylobacter* species and *Rothia mucilaginosa* with a decrease in acetaldehyde-dehydrogenase-producing *Streptococci*. Dysregulation in the xenobiotic metabolism contributes to higher exposure to this carcinogenic metabolite, which promotes the development of oral leukoplakia and its malignant transformation in alcohol consumers.^63^ Areca nut chewers with oral lesions (leukoplakia and submucous fibrosis) had significantly elevated levels of *Oribacterium, Actinomyces*, and *Streptococcus*, including *Streptococcus anginosus*.^64^

In healthy conditions, physical and chemical barriers (e.g., gastric and bile acids) segregate the oral cavity from the gastrointestinal tract. In the absence of these barriers, under pathological conditions, the oral microbiota can translocate to the intestines and modulate the gut microbiota, contributing to the development of gastroenterological diseases and cancer. The oral-gut microbiome axis and its involvement in tumorigenesis gained importance in cancer research.^15^ Oral microorganisms, such as *Fusobacterium, Parvimonas*, and *Peptostreptococcus*, have been found in the intestines of CRC patients, suggesting the role of the oral-gut microbiome axis in colorectal carcinogenesis. *Porphyromonas gingivalis*, a key pathogen in periodontitis, promoted the proliferation of the infected CRC cells with *F.nucleatum*. The salivary microbiota of HCC patients was enriched with *Haemophilus, Porphyromonas* and *Filifactor* species. In mouse models of pancreatic cancer, oral administration of *P. gingivalis* accelerated the progression of pancreatic ductal adenocarcinoma and promoted EMT.^16^ In this study, the normal mucosa adjacent to the carcinoma showed an abundance of Firmicutes in HNSCC patients. In contrast, Bacteroidetes and Firmicutes were abundant in the normal mucosa adjacent to GI carcinoma. There was no significant difference in the prevalence of Bacteroidetes, Firmicutes, Proteobacteria, and Tenericutes between the HNSCC and GIT carcinoma, suggesting overlapping microbial signatures in HNSCC and GIT carcinoma.

Computational approaches are valuable tools for researchers as they provide access to comprehensive data collection and facilitate short-term, cost-effective analyses.^65^ Inherent to any *in silico* study, it has limitations. The alpha and beta diversities of microbial communities involved in carcinogenesis need further exploration. The heterogeneity in microorganisms among varied geographic locations mandates meta-genomics analysis using next-generation sequencing technologies that can demonstrate bacterial profiles and relationships between microbial diversity, genetic variation, and oral diseases.

## Conclusion

Characterization of oral and gastrointestinal microbial dysbiosis might provide new biomarkers useful for diagnosing oral and gastrointestinal carcinomas. While proteomic and genomic biomarkers are subjected to the individual’s biological variations, the oral microbiome is relatively conserved among unrelated individuals. A decrease in Actinobacteria and an enrichment of Flavobacteria at the class level, Neisseriales, Pasteurellales, and Campylobacterales at the order level, *Pasteurellaceae, Flavobacteriaceae, Campylobacteraceae*, and *Peptoniphilaceae* at the family level, and *Hemophilus, Porphyromonas*, and *Leptotrichia* at the genus level were observed in HNSCC compared to the normal mucosa. RICTOR protein, mRNA transcripts such as HIST1H2B, SCARNA11, TBC1D21 gene, and hsa-miR-200a-5p miRNA were significantly correlated with the bacterial species predominant in HNSCC. The oral microbiome and its association with host molecular signatures can serve as tumor biomarkers and oncotherapy targets, enabling early diagnosis and treatment of head and neck cancers.
